# Emerging Roles of IL-33/ST2 Axis in Renal Diseases

**DOI:** 10.3390/ijms18040783

**Published:** 2017-04-07

**Authors:** Wei-Yu Chen, Lung-Chih Li, Jenq-Lin Yang

**Affiliations:** 1 Institute for Translational Research in Biomedicine, Kaohsiung Chang Gung Memorial Hospital, Kaohsiung 833, Taiwan; wychen624@cgmh.org.tw (W.-Y.C.); r5239@cgmh.org.tw (L.-C.L.); 2 Division of Nephrology, Department of Internal Medicine, Kaohsiung Chang Gung Memorial Hospital and Chang Gung University College of Medicine, Kaohsiung 833, Taiwan

**Keywords:** interleukin-33, chronic renal disease, acute renal injury, inflammation

## Abstract

Renal diseases, including acute kidney injury (AKI) and chronic kidney disease (CKD), have a great impact on health care systems worldwide. Similar to cardiovascular diseases, renal diseases are inflammatory diseases involving a variety of cytokines. Primary causes of renal injury include ischemia, uremic toxins, bacteremia, or nephrotoxicity. Inflammation represents an important component following kidney injury. Interleukin (IL)-33 is a member of the IL-1 cytokine family, which is widely expressed in epithelial barrier tissues and endothelial cells, and mediates both tissue inflammation and repair responses. IL-33 is released as a nuclear alarmin in response to tissue damage and triggers innate and adaptive immune responses by binding to its receptor, suppression of tumorigenicity 2 (ST2). Recent evidence from clinical and experimental animal studies indicates that the IL-33/ST2 axis is involved in the pathogenesis of CKD, renal graft injury, systemic lupus nephritis, and AKI. In this review, we discuss the pathological and tissue reparative roles of the IL-33/ST2 pathway in different types of renal diseases.

## 1. Introduction

Renal diseases, including acute kidney injury (AKI) and chronic kidney disease (CKD), are a public health priority and cause a major burden to health care systems worldwide [1,2]. AKI, characterized by sudden loss of kidney function, is a common complication of hospitalization and associates with short-term morbidity and high mortality rates greater than 50% in consequence of commonly association with severe complication [3,4]. Renal interstitial fibrosis and parenchymal tubular cell loss are common outcomes of chronic renal disorders during disease progression [5,6].

Of note, cardiovascular diseases (CVDs) are the leading cause of mortality in patients with renal diseases [7]. CVDs and CKDs share certain risk factors and pathophysiology [8]. Both traditional (diabetes, hypertension and hyperlipidemia) and non-traditional (endothelial dysfunction, oxidative stress and inflammation) risk factors contribute to the initiation and progression of atherosclerotic CVD and CKD. Among these factors, inflammation is an important player involving a variety of chemokines and cytokines.

Cytokines are involved in the progression of renal fibrosis and tissue damage [9]. Pro-inflammatory cytokines (e.g., TNF-α, IFN-γ, IL-1β, IL-6, IL-17, and IL-23) and anti-inflammatory cytokines (e.g., IL-4, TGF-β, and IL-10) expressed by resident and infiltrated immune cells are important mediators for the injury and repair responses [10]. Inflammation [10,11], oxidative stress [12], drug toxicity [13], infection [14], and diabetes [15] are primary factors involved in the pathogenesis of renal injury. In this review, we discuss the pathological and protective roles of the IL-33/ST2 axis involved in CKD and AKI.

### 1.1. Interleukin-33 and ST2 Signaling

**Figure 1:**
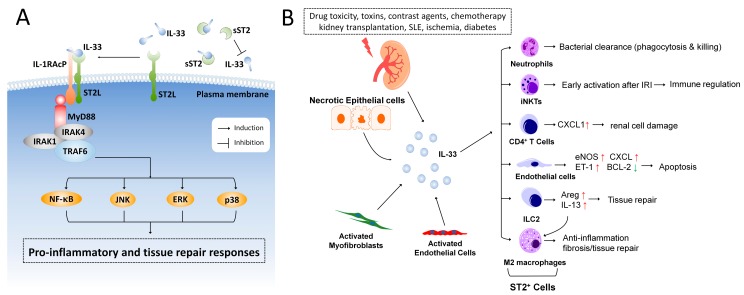
IL-33/ST2 signaling in renal injury. (**A**) IL-33 binds to the receptor complex comprised of ST2L and IL-1RAcP on the cell membrane and induces recruitment of MyD88, IRAK1, IRAK4 and TRAF6, thereby activating the downstream NF-κB, JNK, p38, and ERK pathways. The extracellular soluble form of ST2 (sST2) binds IL-33 as a decoy receptor and has been postulated to be a biomarker in various inflammatory diseases; (**B**) Exogenous factors (such as drug toxicity, toxins, contrast agents, and chemotherapy), kidney transplantation, and diseases associated with nephropathies (SLE, renal ischemia, and diabetes) lead to upregulation of IL-33 in the renal tissues. Upon injury, necrotic epithelial cells, activated myofibroblasts, or endothelial cells release IL-33, triggering an inflammatory response and tissue repair processes by targeting the ST2-expressing cells.

IL-33 was first reported to act as a nuclear factor in high endothelial venules in 2003 [16]. In 2005, Schmitz et al. identified IL-33 as a member of the IL-1 family and a ligand for the orphan receptor, suppression of tumorigenicity 2 (ST2, also known as IL1RL1) [17]. IL-33 functions as a tissue-derived nuclear alarmin [17,18], and is constitutively expressed at high levels in epithelial barrier tissues and endothelial barriers [18,19]. The receptor complex for IL-33 consists of the specific subunit of ST2, encoded by the *IL1RL1* gene, and the coreceptor, IL-1 receptor accessory protein (IL-1RAcP) [18,20]. Two major transcription variants of ST2, the full-length transmembrane form (ST2L) and the soluble form (sST2), have been identified [21]. The sST2 lacks the transmembrane domain and binds to IL-33 as a decoy receptor and has anti-inflammatory properties by regulating IL-33 activity [22,23]. Stimulation of the IL-33 receptor, ST2L, elicits recruitment of MyD88, IRAK1, IRAK4, and TRAF6, then activates downstream NF-κB, c-Jun N-terminal kinases (JNK), p38, and extracellular signal-regulated kinase (ERK) signaling pathways (Figure 1A).

IL-33 was detected in endothelial cells of blood vessels in healthy human tissues [24], but not in mouse tissues [19]. However, IL-33 was upregulated in endothelial cells of inflamed mouse tissues [19,25,26,27]. Apoptotic cells inactivated endogenous IL-33 by caspases to avoid triggering unnecessary immune responses [28,29]. Endogenous IL-33 was constitutively expressed in cell nuclei and was able to associate with chromatin by binding histone H2A/H2B, although its nuclear role still remains unclear [30]. In human endothelial cells, IL-33 likely acts as an extracellular cytokine but not as a nuclear factor in regulating gene expression [31]. Interestingly, nuclear IL-33 was shown to interact with NF-κB and dampen NF-κB-stimulated gene transcription [32]. The nuclear function and stability of IL-33 were regulated by the enzyme ubiquitin-specific protease 17 (USP17) through deubiquitination of IL-33 both at the K48 and K63 sites [33].

Full length IL-33 is released into the extracellular matrix during tissue damage, cell necrosis, or mechanical stress, following exposure to allergens or infection with viruses or parasites [34,35,36]. The full length form of extracellular IL-33 was bioactive; in addition, it can be processed via proteases (cathepsin G and elastase) to become shorter hyperactive forms [23,37]. A recent study reported that IL-33 is susceptible to cysteine oxidation [38]. The biological activity of IL-33 was rapidly inactivated in the extracellular environment by forming two disulfide bonds, resulting in a conformational change and disruption of the ST2 binding site [38]. The release of IL-33 from the nucleus to the extracellular matrix was mediated by a nuclear pore complex in an ATP-dependent manner [21]. Detailed molecular mechanisms involved in regulating IL-33 translocation in living cells remains poorly understood.

The targeted deletion of the nucleus localization domain (amino acid 1–68) of IL-33 led to the spontaneous secretion of the IL-33 protein and triggered a lethal inflammatory response in the IL-33 tm1 knock-in mouse model through ST2-dependent signaling [39]. This suggests that physiological control of IL-33 release is crucial and tightly regulated during tissue homeostasis.

After being released, IL-33 activates various types of immune cells, including neutrophils, eosinophils, mast cells, type 2 helper T cells (Th2), and group 2 innate lymphoid cells (ILC2s), which secrete large amounts of IL-5 and IL-13 for regulation of innate and adaptive immune responses [36,40,41]. IL-33 thus serves as a nuclear alarmin to sense damage and alert adjacent cells and tissues following infection or tissue injury, and therefore has the potential to influence a broad range of diseases [24].

### 1.2. Distribution of IL-33 and ST2 in the Kidney

The distribution and regulation of IL-33 in different tissues and specific cell types have been comprehensively reviewed [42,43,44]. IL-33 is widely expressed in various organs, including heart, brain, kidney, liver, spleen, and lung. At cellular level, IL-33 is predominantly expressed by non-immune cells, including epithelial cells, endothelial cells, and fibroblasts. Immune cells such as activated macrophages were also reported to be sources of IL-33 [45].

Only a few studies have comprehensively investigated the cellular expression of IL-33 and ST2 in kidney tissues following injury. In humans, IL-33 was constitutively expressed and transported to the endothelial nuclei of renal large and small vessels [24]. In mice, IL-33 mRNA is expressed in the kidney tissue [17]. Chen et al. have shown that the IL-33 protein is predominantly distributed in renal tubulointerstitial cells that co-express vimentin and α-smooth muscle actin (α-SMA) by using immunofluorescent staining. The number of IL-33^+^Vimentin^+^ tubulointerstitial cells was markedly increased in the obstructed kidney after unilateral ureteral obstruction (UUO) surgery. CD31^+^ peritubular vascular endothelial cells were relatively minor sources of IL-33 in the control and obstructed kidney [46]. No evident IL-33 expression in glomeruli and tubular epithelial cells was observed in either healthy or obstructed kidney of the UUO mouse model, which suggests that tubulointerstitial cells are major sources of IL-33 in the obstructed kidney [46]. Whether activated immune cells produce IL-33 in the kidney remains to be investigated.

In humans and mice, the transmembrane form ST2L and soluble form sST2 are widely expressed in various organs, including the heart, brain, spleen, lungs, skin, and kidneys [42,43,44]. Endothelial cells [47], cardiomyocytes [26], CD4^+^ T cells [27], macrophages [48], neutrophils, iNKT cells, and ILC2 cells [49,50] were shown to respond to IL-33 activation through their membrane receptor ST2L. The expression of ST2L or IL-33-responsiveness in tubular epithelial cells (including proximal tubular cells, distal tubular cells, collecting duct cells, and pelvic urothelium), mesangial cells or podocytes has not been fully investigated or reported. The major sources of soluble ST2 (sST2) during tissue injury remain largely unknown. A recent study by Zhang et al. demonstrated that intestinal stromal cells and T cells are major sources of sST2 during in mouse model of graft-versus-host disease (GVHD), indicating that T cells are potential sources of circulating sST2 [51]. Endothelial cells and fibroblasts also secrete sST2 in response to activation [52]. In the context of myocardial injury, the sources of circulating serum ST2 are extra-myocardial [53], which indicates that peripheral or systemic inflammation likely attribute to the elevated production of circulation sST2.

Several lines of evidence both from clinical and animal studies demonstrate that the IL-33/ST2 axis is involved in renal diseases. The levels of IL-33 and soluble ST2 are generally tested to evaluate severity and prognosis of renal diseases clinically. Below, we focus on recent findings of the IL-33/ST2 axis involved in renal diseases in human and animal models.

## 2. IL-33 in Chronic Kidney Injury

### 2.1. IL-33 and Chronic Kidney Disease (CKD)

CKD is defined as the presence of renal abnormalities in either function or structure lasting more than three months [54]. Currently serum creatinine, urea nitrogen, and urine analysis are used as biomarkers to monitor renal function [55,56,57]. However, evidence has indicated that these biomarkers are not optimal for detecting early stage CKD. Hence, new potential biomarkers for early CKD have been explored. Bao et al. reported that sST2 serum levels were elevated and associated with disease severity and the level of parathyroid hormone positively correlates with sST2 concentration in non-dialysis CKD patients (stages 2–5) [58]. Patients were divided into three groups according to estimated glomerular filtration rate (group 1: CKD stage 2; group 2: CKD stages 3–4; group 3: CKD stage 5). IL-33 serum levels of CKD patients were not significantly changed in the three groups but serum levels of sST2 strongly correlated with severity of the disease [58]. Another report by Caner et al. demonstrated that IL-33 serum level was not better in early recognition of diabetic nephropathy [59]. The increase in IL-33 serum levels in diabetic nephropathy was not associated with kidney injury, whereas the increase might be a result of diabetes [59]. Musolino et al. reported that reduced IL-33 serum levels in multiple myeloma (MM) patients were associated with a more advanced stage of disease [60]. However, the IL-33 levels in MM patients with kidney failure were not statically significantly different in MM patients who did not have kidney failure [60]. Interestingly, Duan et al. reported that the serum IL-33 expression was predominantly increased in gout patients compared to healthy controls, and the IL-33 levels were higher in gout patients without kidney injury. Furthermore, IL-33 showed a negative correlation with biomarkers of kidney injury, such as creatinine and urea [61]. These findings together indicate that the serum levels of sST2 are more relevant to the progressive deterioration of kidney function. The above clinical studies indicate the serum level of IL-33 may not to be a proper biomarker for kidney failure or CKD.

### 2.2. IL-33 and Diabetic Nephropathy

Diabetic nephropathy (DN) is the most prevalent etiology for CKD with syndromes of progressive increases in excretion of urinary albumin (UAE), elevated blood pressure coupled with glomerular lesions and ultimately functional loss of glomerular filtration leading to renal failure [62]. Inflammatory responses are wildly triggered during DN progression, hence, inflammatory cytokines are profoundly involved in disease progression and severity; IL-33 has emerged as a candidate that is closely involved in the pathogenesis of DN [62]. A recent clinical study by Caner and colleagues investigated the association between IL-33 and DN by analyzing the serum levels of IL-33 from a healthy group, a diabetes mellitus (DM) group without any known renal diseases, and a DM group with microalbuminuria (MA) [59]. The results of Caner’s study demonstrated that IL-33 levels in the DM and DM with MA groups were greater than the healthy group. No significant difference on serum levels of IL-33 was observed between the DM group and the DM with MA group [59]. Therefore, diabetes per se upregulates the serum level of IL-33, but it is not suitable to be used for early diagnosis of DN.

The expression of ST2L, the membrane-bound receptor of IL-33, is upregulated in human diabetic kidney tissues [63] and serum levels of sST2 is elevated in diabetic patients with critical limb ischemia and is directly associated with higher mortality at 1 year after revascularization [64]. The serum level of sST2 was also higher in patients with type 1 diabetes compared to the healthy counterparts [65]. A study by Miller et al. found that elevated serum levels of sST2 was correlated with diabetes but was not related to cardiovascular diseases and atherosclerosis in 639 human subjects [66]. The serum level of sST2, however, has no correlation to smoking, cholesterol, blood pressure, or atheroma (carotid intima media thickness, plaque presence). The results of Miller’s study suggested that sST2 levels are related to diabetes markers in individuals largely without vascular disease [66]. Whether sST2 serum levels correlates with DN requires further investigations.

Patients with diabetic renal disease are more vulnerable to contrast-induced nephropathy [67]. Two animal studies revealed that both serum and renal tissue IL-33 levels are elevated in DM rats with contrast-induced nephropathy [68,69]. Melatonin therapy attenuated kidney IL-33 levels as well as ameliorating contrast-induced kidney injury, which suggests that kidney IL-33 levels may correlate with the degree of kidney injury in this model [68,69].

Taken together, all lines of evidence demonstrate that IL-33 and sST2 are involved in diabetic complications. The role of IL-33 in DM and DM-associated kidney injury remains to be elucidated.

### 2.3. IL-33 and Systemic Lupus Erythematosus (SLE) and Lupus Nephritis

SLE is an autoimmune disease characterized by chronic inflammation involving multiple organ systems [70,71,72]. Lupus nephritis, a serious manifestation of SLE, contributes to the morbidity and mortality in these patients [70,73]. Cytokines and immune cells play a significant roles in the disease pathogenesis of SLE [71]. Deposits of immune complexes and autoantibodies initiate pro-inflammatory responses and leads to glomerular injury of the kidney [74].

Recent studies indicated that the IL-33/ST2 axis had a detrimental effect in the pathogenesis of SLE [75]. Moreover, IL-33 serum levels were increased in SLE patients compared with healthy individuals [76,77]. Another study by Mok et al. has shown that sST2 was significantly increased and positively correlated with SLE disease activity and severity. The sST2 level could be a potential biomarker for diagnosing SLE disease activity; however, the elevated serum sST2 level did not discriminate between active lupus nephritis and non-renal lupus [78]. Moreover, the serum IL-33 levels did not show a significant difference between the control group and SLE patients [78].

In an animal model of SLE, antibody-restrained IL-33 alleviates renal damages, suppresses expansion of Tregs and myeloid-derived Suppressor Cells (MDSC), and prohibits Th17 cells and pro-inflammatory responses, suggesting that blockage of IL-33 has a protective effect on SLE [76]. These results together suggest that IL-33 may be involved in the pathogenesis of SLE. Further investigations on the direct impact of the IL-33/ST2 pathway in lupus nephritis are required.

### 2.4. IL-33 in Renal Cell Carcinoma

Wang and colleagues analyzed the correlation of prognostic significance and tissue IL-33 expression in patients with clear-cell renal cell carcinoma (ccRCC) after surgical resection and found that IL-33 expression was significantly associated with advanced tumor stage [79]. Moreover, IL-33 was suggested as an independent prognostic factor of overall survival for patients with ccRCC after surgery [79].

## 3. IL-33 in Acute Kidney Injury

### 3.1. IL-33 and Acute Kidney Injury (AKI)

AKI is a common complication among hospitalized patients. This disorder, characterized by abrupt deterioration in kidney function and disruption of electrolyte and fluid homeostasis over hours to days, is associated with increased long-term risks of poor outcomes, including CKD, CVD and mortality [1]. An earlier study by Akcay et al. demonstrated that both serum and kidney IL-33 protein levels were upregulated in cisplatin-induced AKI; furthermore, neutralization of IL-33 by administration of sST2 protein reduced infiltrating CD4^+^ T cells and alleviated renal tubular damage [27]. This study indicated that IL-33 promotes AKI through the CD4^+^ T cells/CXCL1 axis.

In the ovalbumin-induced AKI mouse model, anti-IL-33 therapy reduced expression of kidney injury molecule-1, COX-2, iNOS, eNOS, and phosphorylated AMP-activated protein kinase (p-AMPK) in renal parenchyma [80]. Ferrostatin-1, an inhibitor of ferroptosis, was also shown to preserve renal function and prevented the upregulation of IL-33 in folic acid-induced AKI [81]. Kidney IL-33 expression was upregulated by paracetamol-induced nephrotoxicity [82]. Chrysin, a natural antioxidative and anti-inflammatory flavonoid, was shown to inhibit renal damage and decreases renal IL-33 expression in paracetamol-induced kidney injury [82]. These studies together indicate that IL-33 expression in the kidney is upregulated following acute renal injury in different models, and that tissue IL-33 expression possibly correlates with severity of renal damage. Thus, anti-IL-33 therapy is protective and might have beneficial effects in different AKI models [27,80].

### 3.2. IL-33 and Obstructive Renal Injury

Acute and chronic renal injuries lead to the formation of atubular glomeruli, proximal tubular cell loss, immune cells infiltration, collecting ducts remodeling, and interstitial fibrosis [83]. Fibrosis results in accumulation of extracellular matrix proteins with replacement of normal tissue with scar tissue and the fibrotic response is broadly considered irreversible in renal disease [5,6]. The mouse UUO model is widely studied to examine mechanisms of tubulointerstitial fibrosis via surgically induced obstructive renal injury [84]. Our previous study demonstrated that IL-33 is upregulated in the obstructed kidney after UUO [46]. Deficiency of *IL33* reduced renal fibrosis and loss of tubular cells compared with wild type mice [46]. The α-SMA^+^ and vimentin^+^ interstitial myofibroblasts were found to be major sources of IL-33 in the obstructive kidney [46]. Nuclear IL-33 likely functions as a molecular sensor for mechanical stress and the increased level of extracellular ATP, which consequently leads to the translocation of IL-33 to the extracellular matrix [21,85]. In the obstructed kidneys, the extracellular IL-33 possibly promotes renal tissue damage and inflammatory responses. These results suggest that the upregulation of IL-33/ST2 signaling in the obstructed kidney promotes tubular cell injury and interstitial fibrosis [46].

### 3.3. IL-33-Mediated ILC2 Expansion in Acute Renal Injury

In addition to the pro-inflammatory function, IL-33 induces pulmonary tissue repair responses by activating ST2^+^ immune cells, such as group 2 innate lymphoid cells (ILC2s) [86]. Recent studies have shown that ILC2 cells may have protective roles in renal injury as well. Riedel et al. identified the IL-33-responsive ILC2 population in human and mouse kidney [50]. In the model of adriamycin-induced glomerulosclerosis, sustained expansion of ST2^+^ ILC2 by exogenous IL-33 showed ameliorated renal injury. Moreover, eosinophils were required for IL-33-mediated renal protection [50]. Huang et al. demonstrated that IL-25-responsive ILC2 and type 2 multipotent progenitor (MPP^type2^) cells promoted macrophage polarization toward the M2 phenotype in kidney and prevented acute renal ischemia/reperfusion injury [49]. Both studies suggest a protective role of ILC2 in renal protection against adriamycin or ischemia/reperfusion-induced kidney injury. The roles of IL-33 in tissue inflammation and repair by orchestrating immune responses in different models of renal injury will need further comprehensive investigation.

### 3.4. IL-33 and Acute Renal Injury Associated with Infection

Sepsis is a leading cause of mortality in the intensive care unit of hospitals worldwide [87]. An increasing body of evidence indicates that the IL-33/ST2 axis is involved in the initiation and progression of sepsis [88]. IL-33 and sST2 serum levels were elevated in sepsis patients [89,90,91,92]. Infection-induced renal dysfunction and AKI has also been described in moderate-to-severe scrub typhus [93]. The study by Shelite et al. reported that C57BL/6 mice with *Orientia tsutsugamushi* infection had a significant increase in IL-33 expression and ST2L in the kidney and liver. IL33^−/−^ mice showed markedly attenuated endothelial cell apoptosis. Exogenous IL-33 increased disease severity and lethality, which suggests a pathogenic function of IL-33 in *Orientia* infection-triggered EC damage [47].

Interestingly, administration of IL-33 was shown to be protective in mice infected with *Candida albicans* [94]. Tran et al. reported that IL-33 administration enhanced fungal clearance by increasing the phagocytosis activity of neutrophils. Moreover, deletion of *IL13* abolished IL-33-mediated polarization of M2 macrophages and renal functional recovery [48]. This study indicates that therapeutic IL-33 may benefit patients with systemic candidiasis [42]. Taken together, elevation of the IL-33 protein alarms the host tissue and activates innate immune responses to promote bacterial clearance. The IL-33-mediated host responses and its role in infection-associated renal injury appear to be organism- and model-dependent.

## 4. IL-33 in Renal Transplantation

Renal ischemic injury (IRI) is one of the most important issues during kidney transplantation. Thierry and colleagues demonstrated that both serum and urine IL-33 levels were elevated after renal transplantation and determined that invariant natural killer T cells (iNKT) were potential targets of IL-33 in IRI [95]. Zhang et al. studied 64 renal transplant recipients and reported that serum IL-33 was significantly higher and correlated with the elevated production of Th2 cytokines and CD4^+^ T cells in chronic allograft dysfunction (CAD) patients compared to recipients with stable allograft function [96]. A study by Mansell et al. indicated that serum IL-33 level was positively associated with high cardiovascular risk for renal transplant patients. Mansell’s study also found the elevated IL-33 level is associated with age, diabetes, serum phosphorus, microalbuminurea, and diminished GFR, which suggests that IL-33 increases the cardiovascular burden in renal transplant recipients [97].

The serum level of sST2 was found to be significantly increased after kidney transplantation [98], whereas sST2 was unable to induce podocyte injury in vitro or trigger proteinuria in rats, indicating that sST2 was a biomarker of recurrence but not the cause of corticosteroid-resistant idiopathic nephrotic syndrome (INS) [98].

In patients with graft-versus-host disease (GVHD), sST2 was shown to be a biomarker for the development of GVHD as well as correlating with mortality [99]. An elevated IL-33 level was found in nonhematopoietic cells in the gastrointestinal (GI) tract of patients during GVHD [100]. Mice with *ST2* deficiency or dosed with sST2-Fc showed a markedly reduced GVHD lethality [100]. Recently, Zhang et al. demonstrated that intestinal stromal cells and T cells are major sources of sST2 during GVHD. ST2 blockage by a monoclonal antibody reduces sST2-producing T cells while maintaining protective ST2L-expressing T cells during GVHD [51]. The Zhang study provided evidence that blocking IL-33/ST2 signaling is a potential therapeutic target for severe GVHD. Moreover, the study by Matta et al. demonstrated the protective effect of allogeneic hematopoictic cell transplantation (peri-alloHCT) administration of IL-33 and the IL-33-responsive regulatory T cells (Tregs) in mouse models of acute GVHD [101].

The above studies suggest that IL-33 specifically targets ST2-expressing cells such as iNKT cells and Treg cells and functions as a crucial immunomodulator for tolerance induction and suppression of the graft-rejection response in solid organ transplantation [95,101].

## 5. Conclusions

Accumulated evidence indicates that IL-33/ST2 signaling contributes to pathogenesis in multiple diseases that associate with kidney injury. In Table 1, we have summarized recent findings on the IL-33/ST2 axis from clinical and animal studies on kidney diseases. Detection of IL-33 levels in a human fluid specimen largely relies on commercially available ELISA kits. Several studies, however, have demonstrated that the measurement of serum concentrations of IL-33 is challenging and may produce false positive results, which is a caveat for further investigations with serum samples [102,103,104]. The correlation between serum IL-33 levels and impaired renal function remains controversial; nevertheless, upregulation of tissue IL-33 levels is generally consistent in different renal injury models (Table 1).

Exogenous factors (such as drug toxicity, toxins, contrast agents, and chemotherapy), kidney transplantation, and diseases associated with nephropathies (SLE, renal ischemia, and diabetes) lead to upregulation of IL-33 in renal tissue (Figure 1B). After being released, IL-33 elicits both pro-inflammatory and tissue repair responses by activating ST2-expressing cells, including neutrophils, CD4^+^ T cells, iNKT cells, endothelial cells, ILC2s, and polarized M2 macrophages. Whether IL-33 could target other non-immune cells in the kidney, including proximal tubular cells, distal tubular cells, collecting duct cells, urothelial cells, or podocytes is largely unknown and awaits fully investigation.

Upon tissue injury, IL-33 likely functions as a double-edged sword; on one hand IL-33 is critical for tissue repair or elimination of infection while on the other, excessive production can cause tissue and organ damage. Further investigation is necessary to delineate the precise role of the IL-33/ST2 pathway in the regulation of the balance between tissue inflammation and repair. Better understanding of the pathological role of IL-33 in kidney diseases will help in the development of novel therapeutic strategies for treating or preventing kidney diseases.

## Figures and Tables

**Table 1:** Roles of IL-33 and ST2 in renal diseases.

Kidney Diseases	Roles of IL-33/ST2 Axis	IL-33 Levels	sST2 Levels	Species	Reference
Chronic renal failure	sST2 serum levels are elevated and associate with disease severity	(−) Serum	(↑) Serum	human	[58]
	IL-33 serum levels are not correlated with disease severity				
	IL-33 levels are correlated with diabetes but not with renal injury	(−) Serum		human	[59]
Diabetic nephritis	ST2L expression is upregulated in human diabetic kidney tissues	(↑) Kidney mRNA			[63]
	sST2 serum levels are increased in diabetes patients		(↑) Serum	human	[64]
	sST2 serum levels are increased in type 1 diabetes patients		(↑) Serum	human	[65]
Contrast-induced DN	Serum and kidney IL-33 protein levels are elevated in contrast-induced diabetic nephropathy in rats	(↑) Kidney protein		rats	[68,69]
		(↑) Serum			
Multiple myeloma patients with kidney failure	IL-33 serum levels are higher in MM patients	(−) Serum		human	[60]
	No differences in IL-33 levels in MM patients with and without kidney failure				
Gout	IL-33 serum levels are higher in gout patients	(↑) Serum		human	[61]
	IL-33 serum level negatively correlates with biomarkers of kidney injury				
Renal cell carcinoma	IL-33 is associated with advanced tumor stage	(↑) Tissue			[79]
SLE	IL-33 serum levels are elevated in SLE patients	(↑) Serum	(↑) Serum	human mice	[76,77]
	IL-33 inhibition by antibody reduces renal damages and inhibits SLE via expansion of Tregs and MDSC and inhibition of Th17 cells and pro-inflammatory responses			mice	[76]
	sST2 but not IL-33 correlates with disease severity of SLE	(−) Serum	(↑) Serum	human	[78]
UUO	Upregulation of IL-33, sST2, ST2L in obstructed kidney	(↑) Kidney mRNA	(↑) Kidney mRNA	mice	[46]
	*IL-33* deficiency reduces renal fibrosis and tubular loss	(↑) Kidney Protein			
Ischemia/Reperfusion renal injury	ILC2 and MPP ^type2^ cells regulate macrophage phenotype in kidney and prevent acute renal ischemia/reperfusion injury			mice	[49]
Renal Transplantation	Increased serum and urine IL-33 and sST2 levels after renal transplantation	(↑) Urine	(↑) Urine	human	[95]
	IL-33 activates iNKT cells after renal ischemia/reperfusion injury	(↑) Serum	(↑) Serum		
	Serum IL-33 levels are significantly higher in chronic allograft dysfunction	(↑) Serum		human	[96]
	IL-33 levels are positively associated with high cardiovascular risk, diminished eGFR, age, diabetes, serum phosphorus and microalbuminurea for renal transplant recipients	(↑) Serum		human	[97]
	sST2 serum levels are significantly increased after kidney transplantation		(↑) Serum	human	[98]
GVHD	sST2 is a biomarker for the development of GVHD and mortality		(↑) Serum	human	[99]
	Increased IL-33 production is found in nonhematopoietic cells in the gastrointestinal (GI) tract in patients during GVHD	(↑) Serum		mice	[100]
	ST2 blockade by a monoclonal antibody reduces sST2-producing T cells while maintaining protective ST2L-expressing T cells during GVHD		(↑) Serum	mice	[51]
	peri-alloHCT administration of IL-33 expands IL-33-responsive Tregs in mouse models of acute GVHD			mice	[101]
**Drug/toxin-induced AKI**					
Cisplatin	IL-33 protein is induced by cisplatin	(↑) Kidney protein		mice	[27]
	IL-33 exacerbates renal injury via activation CD4^+^ T cells/CXCL1 axis	(↑) Serum			
	Neutralization of IL-33 by sST2 protects against cisplatin-induced AKI				
OVA	Anti-IL-33 therapy reduces KIM-1, COX-2, iNOS, eNOS, and p-AMPK expressions in renal parenchyma.			mice	[80]
Folic acid	Inhibition of ferroptosis preserves renal function and prevents the upregulation of IL-33 in folic acid-induced AKI	(↑) Kidney protein		mice	[81]
		(↑) Serum			
Paracetamol	Upregulation of IL-33 by paracetamol	(↑) Kidney protein		rats	[82]
Adriamycin	ST2^+^ ILC2s are a major ILC population in the human and mouse kidney			mice	[50]
	Exogenous IL-33 ameliorates renal damage by expansion of ST2^+^ ILC2s				
**Infection-associated kidney injury**					
*Orientia tsutsugamushi*	*Orientia *infection increases IL-33 expression and ST2L in the kidney and liver	(↑) Kidney mRNA	(↑) Kidney mRNA	mice	[47]
	*IL33*^−/−^ mice have lower renal cell apoptosis after *Orientia* infection	(↑) Liver mRNA	(↑) Liver mRNA		
	Exogenous IL-33 exacerbates disease and mortality during infection				
*Candida albicans*	IL-33/IL-13 axis enhances fungal clearance by increasing the phagocytosis activity of neutrophils, polarization of M2 macrophages and renal functional recovery			mice	[48]

(↑) : upregulation; (↓): downregulation; (−): no change.
